# Computational Modeling of Human Serum Albumin Binding of Per- and Polyfluoroalkyl Substances Employing QSAR, Read-Across, and Docking

**DOI:** 10.3390/molecules28145375

**Published:** 2023-07-13

**Authors:** Andrea Gallagher, Supratik Kar, Maria S. Sepúlveda

**Affiliations:** 1 Chemometrics and Molecular Modeling Laboratory, Department of Chemistry, Kean University, 1000 Morris Avenue, Union, NJ 07083, USA; gallandr@kean.edu; 2 Department of Forestry and Natural Resources, Purdue University, West Lafayette, IN 47907, USA; mssepulv@purdue.edu; 3 Faculty of Life Sciences, Universidad Andres Bello, Santiago 8370146, Chile

**Keywords:** PFAS, serum albumin, QSAR, read-across, risk assessment, toxicity

## Abstract

Per- and polyfluoroalkyl substances (PFAS) are synthetic chemicals in widespread use that have been shown to be toxic to wildlife and humans. Human serum albumin (HSA) is a known transport protein that binds PFAS at various sites, leading to bioaccumulation and long-term toxicity. In silico tools like quantitative structure-activity relationship (QSAR), read-across, and quantitative read-across structure-property relationship (q-RASPR) are proven techniques for modeling chemical toxicity based on experimental data which can be used to predict the toxicity of untested and new chemicals, while at the same time, help to identify the major features responsible for toxicity. Classification-based and regression-based QSAR models are employed in the present study to predict the binding affinities of 24 PFAS to HSA. Regression-based QSAR models revealed that the packing density index (*PDI*) and quantitative estimation of drug-likeness (*QED*) descriptors were both positively correlated with higher binding affinity, while the classification-based QSAR model showed the average connectivity index of order 4 (*X*4*A*) descriptor was inversely correlated with binding affinity. Whereas molecular docking studies suggested that PFAS with the highest binding affinity to HSA create hydrogen bonds with Arg348 and salt bridges with Arg348 and Arg485, PFAS with lower binding affinity either showed no interactions with either amino acid or only interactions with Arg348. Among the studied PFAS, perfluoroalkyl acids (PFAA) with large carbon chain length (>C10) have one of the lowest binding affinities, compared to PFAA with carbon chain length ranging from 7 to 9, which showed the highest affinity to HSA. Generalized Read-Across (GenRA) was used to predict toxicity outcomes for the top five highest binding affinity PFAS based on 10 structural analogs for each and found that all are predicted as being chronic to sub-chronically toxic to HSA. The developed in silico models presented in this work can provide a framework for designing PFAS alternatives, screening compounds currently in use, and for the study of PFAS mixture toxicity, which is an area of intense research.

## 1. Introduction

Per- and polyfluoroalkyl substances (PFAS) or ‘forever chemicals’ are used in a wide variety of products including household and personal care items, food packaging, fabrics, and manufacturing and chemical facilities [1]. PFAS are persistent and do not easily degrade under environmental conditions, leading to bioaccumulation [2,3]. In humans, PFAS exposure has been linked to thyroid disruption, cancer, low birth weight, suppressed vaccine response, obesity, liver disease, kidney disease, and cardiovascular disease, among other adverse health outcomes [1,4,5].

PFAS bind to proteins and this interaction has been postulated to play a major role in bioaccumulation and toxicity. Human serum albumin (HSA) is the primary blood transport protein and has been shown to bind to PFAS at various sites; therefore, serves as a major transport protein for the distribution and bioaccumulation of PFAS. Studies have been conducted investigating the chemical properties of PFAS that affect binding affinity to HSA. A study by Allendorf et al. [6] showed that the albumin/water partition coefficients for several perfluoroalkyl acids (PFAA) were positively correlated with increasing chain length, and absorption was higher for PFAS with sulfonate groups compared to carboxylate groups. Jackson et al. [7] found that PFAS with 6–8 carbon chain length had the highest binding affinity with HSA. In a study by Chi et al. [8], perfluorooctane sulfonic acid (PFOS) and perfluorooctanoic acid (PFOA) both bound to HSA, with PFOS having a stronger binding affinity due to its sulfonate group. Delva-Wiley et al. [9] found that GenX bound HSA at multiple sites, each site involving a hydrogen bond with an arginine residue. Hydrophobicity was a crucial factor for PFAS binding to bovine serum albumin, which is structurally similar to HSA [10].

In sum, the major mechanisms by which HSA interacts with PFAS at the active site involves hydrophobic interactions with hydrophobic pockets and electrostatic interactions with charged amino acid residues. This binding could displace endogenous ligands, such as fatty acids, affecting binding affinity and influencing the transport of other molecules and metabolites. In addition, binding of PFAS to HSA could lead to structural changes in the protein impacting its function. Therefore, additional studies that further characterize the interaction of PFAS with HSA are needed.

In silico models are a proven method to predict toxicity as well as evaluate the mode of action (MOA) action for a specific endpoint. In our previous work, we employed quantitative structure-activity relationships (QSAR) and docking methods to explore the endocrine-disrupting activity of PFAS [11]. In another study, QSAR was employed to evaluate the change in toxicity among single, binary, and tertiary PFAS mixtures, followed by understanding the MOA of their toxicity in terms of synergism and/or additivity for mixtures [12]. The experimental study suggested that PFOS was the most cytotoxic and perfluorohexane sulfonate (PFHxS) the least cytotoxic among the four studied PFAS. In conclusion, experimental and modeling outcomes confirmed that mixtures were roughly additive, with the exception of PFOS and PFOA, which were found to be weakly synergistic.

The objective of this study was to model the binding affinity of 24 PFAS with HSA. Classification- and regression-based QSAR, followed by read-across models and docking studies, were used to model and predict the toxicity and potential adverse effects to HSA, followed by a description of major structural and physicochemical features, driving binding affinities to HSA. The developed in silico toxicity models also can be used in the early stages of PFAS development to prioritize compounds for further testing, reducing the time and cost associated with experimental testing, followed by offering insights into underlying mechanisms of toxicity, and support decision-making processes in various industries as well as in environmental risk assessment. Importantly, our approach can also be used to study PFAS interactions and aid in the understanding of PFAS mixture toxicity.

## 2. Results and Discussion

### 2.1. Classification-Based QSAR Model

After combining training and test data sets, the model classified 11 PFAS as having ‘high’ (H) binding affinity and 13 PFAS as having ‘low’ (L) binding affinity with HSA. Of the 16 PFAS in the training set, 9 were classified as having H binding affinity and 7 were classified as having L binding affinity. Among the remaining 8 PFAS in the test set, 4 were classified as having H binding affinity and 4 were classified as having L binding affinity (Table 1). The discriminant function Δ*P* is represented by the following Equation (1):

(1) $$\Delta P=-14.668+33.651\times Eig12\_AEA\left(bo\right)+0.378\times DECC+30.405\times X4A\;(Wilks^{\prime }\;\lambda =0.26,\;F\left(3,12\right)=10.45,\;P<0.001$$

Detailed validation metrics results are illustrated in Table 2. Values above the potency threshold are classified as L and values below the potency threshold are classified as H. As all three descriptors in the equation contribute positively, higher values for these descriptors will most likely result in L classification and lower values are more likely to result in H classification. As can be seen in the equation, *X*4*A* has the highest contribution, meaning it is the most important factor in determining the binding affinity in this model. *X*4*A* refers to the average connectivity index of order 4, while *Eig*12_*AEA*(*bo*) refers to the eigenvalue number12 from the augmented edge adjacency matrix weighted by bond order and *DECC* refers to eccentric topological indices.

**Figure 1:**
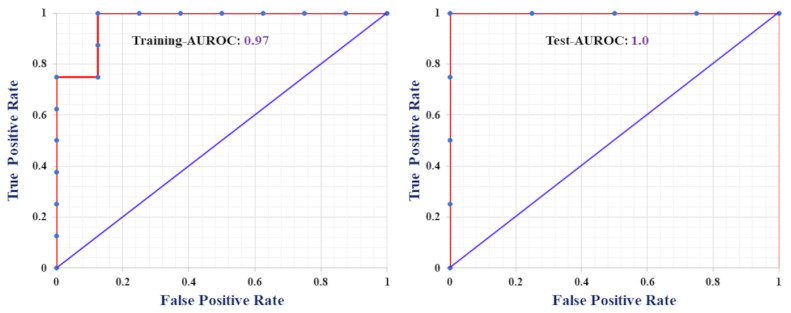
ROC curve for training and test sets based on the developed classification-based model.

**Figure 2:**
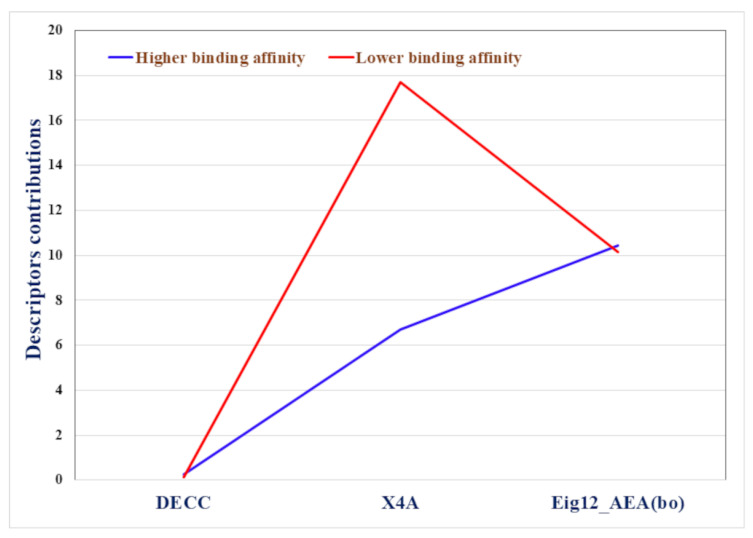
Contribution plot for the modeled features indices to the discriminant functions for higher and lower binding affinity groups of PFAS to HSA.

The receiver operating characteristic (ROC) curves in Figure 1 illustrate the perfect classification ability of the developed model (Equation (1)), where training and test ROC curves achieved 0.97 and 1 value, respectively. Examining the contribution plot in Figure 2, we are certain that *X*4*A* is the most important discriminating feature between higher and lower binding affinity of PFAS towards HSA. From the plot, it is quite evident that the higher the *X*4*A* value of a PFAS, the lower the binding affinity. Importantly, an additional two features: *Eig*12_*AEA*(*bo*) and *DECC* are equally important for the development of the model, but considering the discrimination between H and L binding groups, they don’t have much clear distinction.

### 2.2. Regression-Based ‘Small Dataset QSAR’ Model for Undivided Dataset

Two PFAS had no binding affinity values; therefore, for the development of the regression-based QSAR model, we considered only 22 PFAS. As the number is quite low, we decided to take the whole dataset for modeling purposes employing ‘SmallDataModeler’, which is an approved modeling tool when there is not enough data to utilize training and test data sets separately. We developed two models employing multiple linear regression (MLR) and partial least squares (PLS) chemometric tools. Based on the goodness-of-fit and internal validation metrics, it was determined that the PLS model (Model 2) was slightly better than the MLR model (Model 1). The quality of both models is depicted in Table 3.

The developed equation for Model 2 is as follows:

(2) $$EC_{50}\left(\mathrm{mM}\right)=24.427-23.551\times PDI-0.862\times GATS8v-0.607\times MATS8m-4.388\times QED$$

The regression-based Small Dataset MLR model consists of 4 descriptors: *MATS*8*m*, *GATS*8*v*, *PDI*, and *QED*. The *PDI* descriptor refers to packing density index, defined as the ratio between the McGowan volume (*Vx*) and the total surface area from P_VSA-like descriptors (*SAtot*), or $PDI=\frac{Vx}{SAtot}$. The *MATS*8*m* descriptor refers to Moran autocorrelation of lag 8 weighted by mass. The *GATS*8*v* descriptor refers to Geary autocorrelation of lag 8 weighted by van der Waals volume. The *QED* descriptor refers to the quantitative estimation of drug-likeness. This descriptor is based on 8 other molecular properties, which are used to obtain a set of desirability functions through asymmetric double sigmoidal (ADS) functions. *QED* is defined by the equation $QED=e^{\frac{\sum_{i=1}^{n}w_{i}\ln d_{i}}{\sum_{i=1}^{n}w_{i}}}$, where *d_i_* is the desirability of the property, *w_i_* is the weight applied to each function, and n is the number of desirability functions.

**Figure 3:**
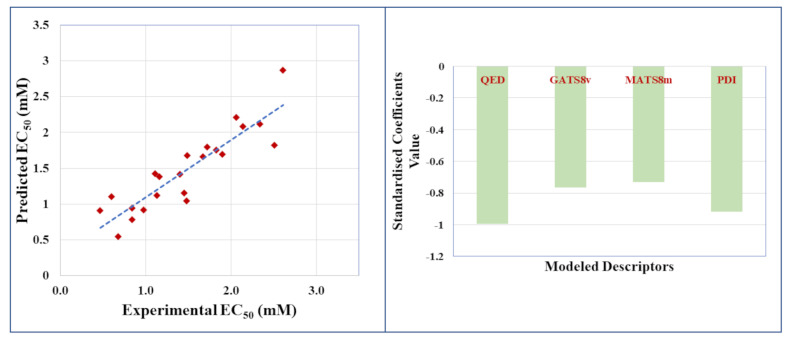
Scatter plot and variable significance plot for the best regression-based QSAR model (PLS model: Model 2).

All four descriptors in the equation have a negative contribution to the equation, meaning that higher values for each descriptor will lead to a lower *EC*_50_ or higher binding affinity. To obtain a PFAS with low binding affinity to HSA and, therefore, lower toxicity, ideally the values for all four descriptors should be low, resulting in a more positive *EC*_50_. The scatter plot employing the best PLS-based model in Figure 3 (Left) shows that experimental binding affinities in terms of *EC*_50_ are well correlated with the predicted affinities. The scatter plot shows that the points fell close to the line of perfect fit, which further supports the predictive efficacy of the developed QSAR model. The variable significance plot in Figure 3 (Right) displays the standardized values for each descriptor of the PLS-based equation. All four descriptors negatively contribute to the equation, with *QED* showing the highest contribution followed by *PDI*, *GATS*8*v*, and *MATS*8*m* to the model.

### 2.3. Read-Across Results

**Figure 4:**
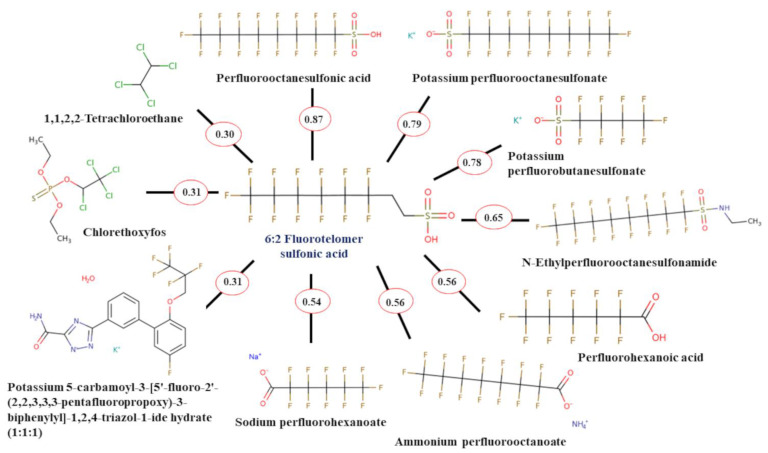
Top 10 ToxPrint Chemotype analogues for 6:2 fluorotelomer sulfonic acid.

Based on the top 10 ToxPrint Chemotype analogues for each PFAS, GenRA predicted the toxic effects of S5, C6, C4, C5, and C12 (top five highest binding affinity to HSA in the present study). Notably, all 5 PFAS were predicted to be sub-chronically toxic to albumin (ACT scores of 1, 0.818, 0.812, 0.812, and 0.816, respectively), with S5 and C12 having ACT scores of 1 and 1, respectively were also predicted to be chronically toxic. However, it should be noted that the predictions were validated in combination with AUC values and *p*-values. All AUC values for each prediction were 0, and *p*-values ranged from 0.73 to 1. Ideally, the AUC should be greater than 0.7 and *p*-value should be less than 0.1. While the predictions have high ACT scores, the validation metrics show that the predictions are not that reliable due to low AUC and high *p*-values. One of the major reasons is we are just challenged by the coverage of PFAS toxicity data and the extent to which we can quantify the performance for target chemicals with any degree of robustness. Figure 4 depicts the top 10 ToxPrint Chemotype analogues for 6:2 fluorotelomer sulfonic acid (6:2 FTSA), the PFAS with the highest binding affinity to HSA in the studied dataset. The top 10 ToxPrint Chemotype analogues for the remaining top four PFAS are shown in Supplementary Materials.

Four of the structural analogues were common between all five molecules and contributed to the albumin toxicity prediction with known data. Potassium perfluorobutanesulfonate, an analogue for all five molecules, had subchronic toxic albumin effects at 600 mg/kg/day. N-Ethylperfluorooctanesulfonamide, an analogue for all five molecules, had subchronic toxic albumin effects at 10.1 mg/kg/day. 1,1,2,2-Tetrachloroethane, an analogue for S5, C6, C4, and C5, had subchronic toxic albumin effects at 40 mg/kg/day. Chlorethoxyfos, an analogue for S5 and C12, had subchronic toxic albumin effects at 1.25 mg/kg/day and chronic toxic albumin effects at 1.86 mg/kg/day. Toxicity data and predictions for all five sets of GenRA can be viewed in Supplementary Materials.

### 2.4. Docking Results

**Figure 5:**
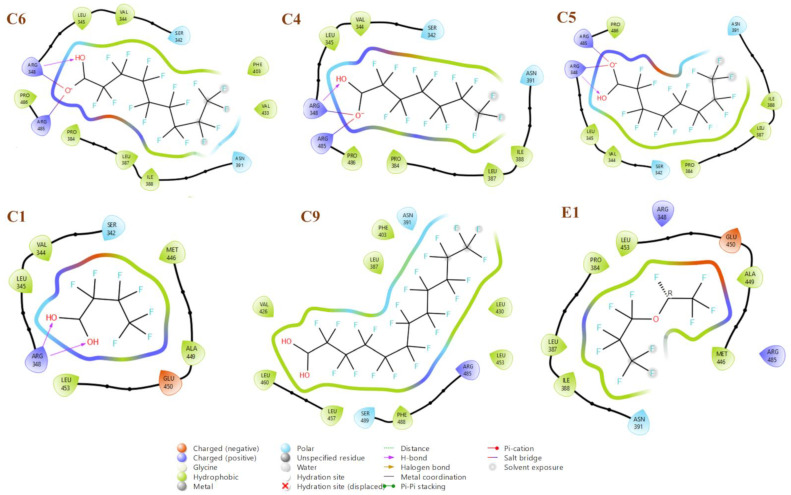
Amino acid interactions of PFAS showing the highest and lowest binding affinities to HSA.

Docking interaction diagrams for the three highest binding affinities (C6, C4, and C5) and three lowest binding affinities (C1, C9, and E1) show the crucial amino acids involved in binding (Figure 5). Interestingly, all three of the highest affinity PFAS form a hydrogen bond with Arg348 and two salt bridges between Arg485 and Arg348, as well as some hydrophobic and polar interactions. In contrast, C9 and E1 do not form any hydrogen bonds or salt bridges, only hydrophobic and polar interactions, leading to their lowest binding affinity to HSA among the studied PFAS. C1 forms two hydrogen bonds with Arg348, but no salt bridges, indicating that the salt bridges are essential to high binding affinity to HSA. It is interesting to note that despite C9 containing a carboxylate group like C4, C5 and C6, it still has one of the lowest binding affinities, likely due to its large carbon chain length (>C10), which is corroborated by our earlier work [11]. PFAA with carbon chain length ranging from 7 to 9 (C4, C5 and C6) show the highest affinity to HSA.

## 3. Materials and Methods

### 3.1. Dataset

Binding affinities of PFAS to HSA in terms of half maximal effective concentrations (*EC*_50_ in mM) for 24 diverse PFAS including perfluoroalkyl sulfonic acids (C4–C8), perfluoroalkyl carboxylic acids (C4–C12), mono- and polyether perfluoroalkyl ether acids, and polyfluoroalkyl fluorotelomer were collected from the literature [7]. Out of the 24 PFAS, *EC*_50_ were determined for 22 PFAS. The *EC*_50_ values were obtained from the concentration-response curves using a 4-parameter variable slope model. *EC*_50_ quantifies binding affinity, specifically quantifies the concentration of ligand at which half of the target protein is bound. *EC*_50_ also measures the concentration of ligand necessary to induce half of the maximum possible effect. Out of the 24 PFAS, the two fluorotelomer alcohols (4:2 FTOH and 6:2 FTOH) did not bind to PFAS as per experimental study; therefore, they can be classified as ‘non-toxic’ among the studied PFAS. Thus, all 24 PFAS were used in the development of a classification-based QSAR model and 22 PFAS were used in the regression-based QSAR model (Table 1). PFAS with *EC*_50_ of 1.45 mM or lower were classified as having H binding affinity to HSA, while the rest were classified as L binding affinity to HSA including the 2 fluorotelomer alcohols with no interactions for the classification QSAR.

### 3.2. Descriptor Calculation

The chemical structures for the 24 PFAS were uploaded in alvaDesc 2.0.16 [13] for the descriptors calculation. For the classification-based QSAR model, a pool of 753 2D descriptors were calculated for the 24 PFAS. For the regression-based QSAR model, a pool of 736 2D descriptors were calculated as 4:2 FTOH and 6:2 FTOH were excluded due to non-interactions with HSA. Both descriptor pools included the following types of descriptor classes: constitutional indices, ring descriptors, topological indices, walk and path counts, connectivity indices, information indices, 2D matrix-based descriptors, 2D autocorrelations, Burden eigenvalues, P_VSA-like descriptors, ETA indices, drug-like indices, MDE descriptors, chirality descriptors, atom-centered fragments, atom-type E-state indices, charge descriptors, edge adjacency indices, functional group counts, molecular properties, pharmacophore descriptors, and 2D atom pairs.

### 3.3. QSAR Modeling

The classification-based QSAR model was developed to identify major discriminatory features between the higher to lower binding affinity classes of PFAS, using genetic algorithm-linear discriminant analysis (GA-LDA) with the ‘ClassificationBasedQSAR v1.0.0′ tool by DTC Lab Software [14]. The classification dataset was divided into a training and a test set for modeling with random method at a ratio of 2:1, which resulted in n_test_ = 8 and n_training_ = 16. Due to the small number of data points, in case of the regression-based QSAR modeling, the ‘Small Dataset Modeler’ tool from DTC Lab Software [14] was used to utilize the whole data set for the exhaustive double cross-validation approach, which does not require dataset division. SmallDataModeler v1.0 by DTC Lab Software was employed using 3 compounds in the validation set by genetic algorithm-multiple linear regression (GA-MLR) selection. The SmallDataModeler employs an exhaustive double cross-validation approach and a set of optimal model selection techniques including consensus predictions for performing the small-dataset QSAR modelling. It performs four basic steps, i.e., (i) data pre-treatment, (ii) model development using exhaustive double cross-validation approach, (iii) selection of optimal model, and (iv) model validation (both internal and external).

### 3.4. Validation, Applicability Domain, and Randomization

Accuracy, Sensitivity, Specificity, Precision and F-measure, geometric means (*G-means*), *Cohen’s* *kappa*, Matthew’s correlation coefficient (*MCC*), and receiver operating characteristic (ROC) were evaluated as validation metrics for classification-based QSAR [15]. The area under the ROC curve (AUROC) evaluates the performance of a diagnostic variable, where an AUROC of 1 is ideal and an AUROC of 0.5 indicates a random guess, while the regression-based QSAR model and q-RASAR models were validated using goodness-of-fit (*R*^2^), internal validation metric leave-one-out cross-validation (*Q*^2^*_LOO_*), *r_m_*^2^ metrics, and mean absolute error (*MAE*_(95%)_). The mathematical definition of major statistical metrics is listed in Table 4.

Here, *W_g_* = cross-product matrix for within group variance, *B_g_* = cross-product matrix for between group variance, *σ*_1_ is the standard deviation of population 1, *s*_1_ is the standard deviation of the sample drawn from population 1, *σ*_2_ is the standard deviation of population 2, *s*_2_ is the standard deviation of the sample drawn from population 2, *λ_i_* = eigen value, *det* = determinant of a matrix, *TP* = true positive, *FN* = false negative, *TN* = true negative, *FP* = false positive, *λ* = *Wilk’s* lambda; $P_{r}\left(a\right)$: relative observed agreement between the predicted classification of the model and the known classification; $P_{r}\left(e\right)$: hypothetical probability of chance agreement, *Y_obs_* = observed response, *Y_calc_* = calculated response, $\bar{Y}$ = mean response of the analyzed set (any), ${\bar{Y}}_{training}$ = mean response of training set, *Y_obs(training)_* = observed response of the training set, *Y_pred(training)_* = predicted response of the training set, *r*^2^ = squared correlation coefficient, and *r*_0_^2^ = squared correlation coefficient with zero intercept.

### 3.5. Docking Study

Molecular docking was performed to identify important interactions between the 24 PFAS under study and the protein structure of HSA in complex with PFOA (PDB: 7AAI). The ligands and protein were prepared with the LigPrep [16] and Protein Preparation tools on Maestro module of Schrodinger 2023. The Receptor Grid Generation tool generated a grid file around the top docking site, with an enclosed size of 20 Å. The ligands were docked using the extra precision (XP) Glide module [17] of Schrodinger 2023. The docking method was validated by redocking the co-crystallized ligand PFOA and calculating the RMSD between the docked ligand and the original, which was 0.092 Å. Once the docking protocol and binding active site were validated, we docked all 24 PFAS. Later glide docking energies were considered for correlations between the binding affinity of PFAS to HSA.

### 3.6. Read-Across

**Figure 6:**
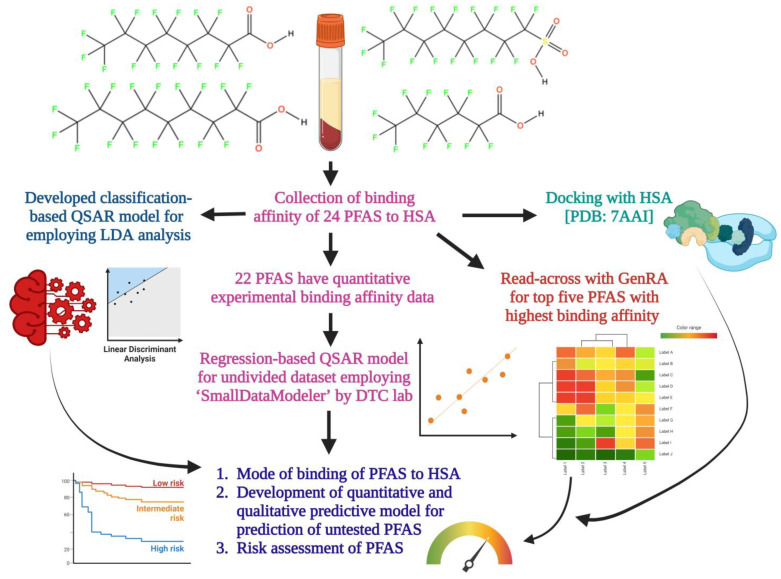
Flowchart of the employed computational approach to execute the study. The figure was created with ‘BioRender.com’ (accessed on 2 July 2023).

Chemical read-across (RA) is one of the major in silico approaches to fill data gaps when there is not enough data available. The available data of a particular substance (referred to as the source) are utilized to forecast the corresponding endpoint(s) for another substance (known as the target) that lacks data but is deemed ‘similar’ in certain aspects, such as structural similarity among chemicals. A disadvantage of the RA approach is that it is subjective and driven by an expert’s specific approach, which can lead to issues of reproducibility and scalability. An alternative to RA is Generalized Read-Across (GenRA) [18], which is an algorithmic developed by the US EPA that enables more objective and reproducible predictions of in vivo toxicity and in vitro bioactivity. In the present study, we used GenRA to predict the toxicity effects of the top five highly bound PFAS to HSA from the experimental data: S5, C6, C4, C5, and C12. For each molecule, the top 10 structural analogues were identified with pairwise similarity metrics, based on ToxPrint Chemotypes. A data matrix was generated for each set of the 10 analogues, displaying toxicity effects from available data. This approach predicted the toxicity effects of each of the five molecules under various categories including chronic, multigenerational, developmental, subacute, and subchronic effects. Based on the GenRA, we predicted a total of 307 chronic and subchronic toxicity endpoints for HSA resulting from PFAS. Results were further validated by similarity-weighted activity scores (ACT), AUC, and *p*-values. The complete workflow employed in the present study is depicted in Figure 6.

## 4. Overview and Conclusions

We have employed multiple in silico modeling approaches like QSAR, RA, and docking to model HSA binding affinity of PFAS. We found that PFAS with long carbon chains (>C10) have lower binding affinities with HSA compared to shorter chain PFAS (C7 to C9), which interacted with HSA with the highest affinity. The RA study also predicted and confirmed that the top five highest binding affinity PFAS as per studied data are also chronic to sub-chronically toxic to HSA. The developed models are not only important to predict the binding affinities of the 25 PFAS tested, but also can be efficiently employed to predict new and untested PFAS’ binding to HSA considering applicability domain in mind. The docking study also offered major insights on amino acid interactions between PFAS and HSA, which will aid in the identification of potentially hazardous PFAS. Additionally, these models can be used to evaluate the interactions of PFAS mixtures, which is what humans and other biota are exposed to in the real world, and is an area of intense research.

## Figures and Tables

**Table 1:** Modeled PFAS followed by experimental and predicted results obtained from classification and PLS-based regression QSAR.

ID	CAS	Chemical Name	Regression-Based QSAR		Classification-Based QSAR		Docking
			Observed *EC*_50_ (mM)	Predicted *EC*_50_ (PLS Model)	Observed Classification	Predicted Classification (LDA Model)	Glide Energy (kcal/mol)
C1	375-22-4	Perfluorobutanoic acid (PFBA)	2.61	2.87	L	L	−19.583
C2	2706-90-3	Perfluoropentanoic acid (PFPeA)	2.14	2.09	L	L	−21.171
C3	307-24-4	Perfluorohexanoic acid (PFHxA)	1.40	1.41	L	L	−20.473
C4	375-85-9	Perfluoroheptanoic acid (PFHpA)	0.68	0.55	H	H	−31.114
C5 *	335-67-1	Perfluorooctanoic acid (PFOA)	0.84	0.78	H	H	−33.059
C6	375-95-1	Perfluorononanoic acid (PFNA)	0.60	1.10	H	H	−37.045
C7 *	335-76-2	Perfluorodecanoic acid (PFDA)	1.11	1.42	H	H	−40.381
C8	2058-94-8	Perfluoroundecanoic acid (PFUnDA)	1.49	1.68	H	H	−35.144
C9	307-55-1	Perfluorododecanoic acid (PFDoA)	2.51	1.83	L	L	−25.948
C10	356-02-5	3:3 Fluorotelomer carboxylic acid (3:3 FTCA)	2.06	2.21	L	L	−22.287
C11	914637-49-3	5:3 Fluorotelomer carboxylic acid (5:3 FTCA)	1.48	1.04	H	H	−25.859
C12	27854-30-4	6:3 Fluorotelomer carboxylic acid (6:3 FTCA)	0.84	0.95	H	H	−27.848
C13	34598-33-9	8:3 Fluorotelomer carboxylic acid (8:3 FTCA)	1.16	1.38	H	H	−29.169
E1 *	3330-15-2	Heptafluoropropyl 1,2,2,2-tetrafluoroethyl ether (E1)	2.34	2.11	L	L	−18.76
E2	13252-13-6	2,3,3,3-Tetrafluoro-2-(heptafluoropropoxy)propanoic acid (HFPO-DA)	1.83	1.75	L	L	−24.764
E3	749836-20-2	7H-Perfluoro-4-methyl-3,6-dioxaoctanesulfonic acid (Nafion BP2)	1.90	1.70	L	L	−27.534
E4 *	151772-59-7	Perfluoro-3,6,9-trioxadecanoic acid (PFO3DoDA)	1.67	1.66	L	L	−34.337
S1 *	375-73-5	Perfluorobutanesulfonic acid (PFBS)	1.72	1.79	L	L	−26.738
S2 *	355-46-4	Perfluorohexanesulfonic acid (PFHxS)	0.98	0.92	H	H	−27.822
S3 *	1763-23-1	Perfluorooctanesulfonic acid (PFOS)	1.13	1.12	H	H	−31.682
S4	757124-72-4	4:2 Fluorotelomer sulfonic acid (4:2 FTSA)	1.45	1.16	H	L	−23.829
S5	59587-38-1	6:2 Fluorotelomer sulfonic acid (6:2 FTSA)	0.47	0.91	H	H	−28.131
O1	2043-47-2	4:2 Fluorotelomer alcohol (4:2 FTOH)	N/A	N/A	L	L	−16.373
O2 *	647-42-7	6:2 Fluorotelomer alcohol (6:2 FTOH)	N/A	N/A	L	L	−21.834

* Denotes compounds in test set for classification-based QSAR model.

**Table 2:** Qualitative prediction of classification-based QSAR model.

Metrics	Training Set	Test Set
*Sensitivity* (%)	87.5	100
*Specificity* (%)	100	100
*Precision* (%)	100	100
*Accuracy* (%)	93.75	100
*F-measure* (%)	93.33	100
*MCC*	0.88	1
AUROC	0.97	1
*Cohen’s κ*	0.88	1
*G-means*	93.5	100

**Table 3:** Quality of regression-based ‘Small Dataset QSAR’ models.

Model	Chemometric Tool	No. of Descriptors	LV	*R* ^2^	*Q* ^2^_(*LOO*)_	$\bar{r_{m\left(\mathrm{LOO}\right)}^{2}}\;$	MAE_(95%)_
1	MLR	4	-	0.805	0.677	0.588	0.221
2	PLS	4	3	0.802	0.691	0.594	0.205

LV: Latent variable.

**Table 4:** Mathematical formula of statistical validation metrics employed in the present classification- and regression-based QSAR models.

Metrics Defining Statistical Quality of the Classification-Based QSAR Models		
Sl. No.	Mathematical Definition	
1	$\lambda =det\left(\frac{W_{g}}{B_{g}+W_{g}}\right)$	Goodness-of-fit and quality measures
2	F=S12σ12S22σ22	
3	$Sensitivity=\frac{TP}{TP+FN}$	Internal and external validation metrics and parameters for ROC analysis
4	$Specificity=\frac{TN}{TN+FP}$	
5	$Precision=\frac{TP}{TP+FP}$	
6	$Accuracy=\frac{TP+TN}{TP+FN+TN+FP}$	
7	$F-measure\left(\%\right)=\frac{2}{\frac{1}{Precision}+\frac{1}{Sensitivity}}$	
8	$MCC=\frac{\left(TP\times TN\right)-\left(FP\times FN\right)}{\sqrt{\left(TP+FP\right)\times \left(TP+FN\right)\times \left(TN+FP\right)\times \left(TN+FN\right)}}$	
9	$G-means=\sqrt{Specificity\times Sensitivity}$	
10	$P_{r}\left(a\right)=\frac{\left(TP+TN\right)}{\left(TP+FP+TN+FN\right)}$ $P_{r}\left(e\right)=\frac{\left\{\left(TP+FP\right)\times \left(TP+FN\right)\right\}+\left\{\left(TN+FP\right)\times \left(TN+FN\right)\right\}}{{\left(TP+FN+FP+TN\right)}^{2}}$ $Cohen^{\prime }s\;K=\frac{P_{r}\left(a\right)-P_{r}\left(e\right)}{1-P_{r}\left(e\right)}$	
**Metrics defining statistical quality of the regression-based models**		
**Sl. No.**	**Mathematical definition**	
11	$R^{2}=1-\frac{\sum {\left(Y_{obs}-Y_{pred}\right)}^{2}}{\sum {\left(Y_{obs}-\bar{Y_{training}}\right)}^{2}}$	Goodness-of-fit and quality measures
12	$Q_{LOO}^{2}=1-\frac{\sum {\left(Y_{obs\left(training\right)}-Y_{pred\left(training\right)}\right)}^{2}}{\sum {\left(Y_{obs\left(training\right)}-\bar{Y_{training}}\right)}^{2}}$	Internal parameters For robustness checking
13	Mean absolute error $MAE=\frac{1}{n}\times \sum \left|Y_{obs}-Y_{pred}\right|$	Prediction error
14	*r_m_^2^ metric* $\bar{r_{m}^{2}}=\frac{r_{m}^{2}+r\prime_{m}^{2}}{2}\;\;\;\mathrm{and}\;\;\;\Delta r_{m}^{2}=\left|r_{m}^{2}-{r^{\prime }}_{m}^{2}\right|$ where $r_{m}^{2}=r^{2}\times (1-\sqrt{r^{2}-r_{0}^{2}}$) The parameters *r*^2^ and *r_0_*^2^ are defined as follows: $r_{0}^{2}=1-\frac{\sum {\left(Y_{obs}-k\times Y_{pred}\right)}^{2}}{\sum {\left(Y_{obs}-{\bar{Y}}_{obs}\right)}^{2}}$& $r_{0}^{\prime 2}=1-\frac{\sum {\left(Y_{pred\;}-k^{\prime }\times Y_{obs\;}\right)}^{2}}{\sum {\left(Y_{pred\;}-{\bar{Y}}_{pred\;}\right)}^{2}}$ The terms *k* and *k’* are defined as: $k=\frac{\sum \left(Y_{obs}\times Y_{pred\;}\right)}{\sum {\left(Y_{pred\;}\right)}^{2}}$ & $k^{\prime }=\frac{\sum \left(Y_{obs}\times Y_{pred}\right)}{\sum {\left(Y_{obs}\right)}^{2}}$ The *Y_obs_* and *Y_pred_* values have been scaled at the beginning using the following formula: $Y_{i(scaled)}=\frac{Y_{i}-Y_{\min(obs)}}{Y_{\max(obs)}-Y_{\min(obs)}}$	Scaled *r_m_*^2^ metrics for internal predictivity

## Data Availability

Supporting data can be found under Supplementary Materials.

## Supplementary Materials

The following supporting information can be downloaded at: https://www.mdpi.com/article/10.3390/molecules28145375/s1.

## References

[1] EPA (2022). Our Current Understanding of the Human Health and Environmental Risks of PFAS.

[2] Ahrens L. (2011). Polyfluoroalkyl compounds in the aquatic environment: A review of their occurrence and fate. J. Environ. Monit..

[3] Lassen C., Jensen A.A., Potrykus A., Christensen F., Kjølholt J., Jeppesen C.N., Mikkelsen S.H., Innanen S. (2013). Survey of PFOS, PFOA and Other Perfluoroalkyl and Polyfluoroalkyl Substances. https://www2.mst.dk/udgiv/publications/2013/04/978-87-93026-03-2.pdf.

[4] National Toxicology Program (NTP) (2016). Immunotoxicity Associated with Exposure to Perfluorooctanoic Acid (PFOA) or Perfluorooctane Sulfonate (PFOS). https://ntp.niehs.nih.gov/go/749926.

[5] Fenton S.E., Ducatman A., Boobis A., DeWitt J.C., Lau C., Ng C., Smith J.S., Roberts S.M. (2021). Per- and polyfluoroalkyl substance toxicity and human health review: Current state of knowledge and strategies for informing future research. Environ. Toxicol. Chem..

[6] Allendorf F., Berger U., Goss K.-U., Ulrich N. (2019). Partition coefficients of four perfluoroalkyl acid alternatives between bovine serum albumin (BSA) and water in comparison to ten classical perfluoroalkyl acids. Environ. Sci. Process. Impacts.

[7] Jackson T.W., Scheibly C.M., Polera M.E., Belcher S.M. (2021). Rapid Characterization of human serum albumin binding for per- and polyfluoroalkyl substances using differential scanning fluorimetry. Environ. Sci. Technol..

[8] Chi Q., Li Z., Huang J., Ma J., Wang X. (2018). Interactions of perfluorooctanoic acid and perfluorooctanesulfonic acid with serum albumins by native mass spectrometry, fluorescence and molecular docking. Chemosphere.

[9] Delva-Wiley J., Jahan I., Newman R.H., Zhang L., Dong M. (2021). Computational analysis of the binding mechanism of GenX and HSA. ACS Omega.

[10] Alesio J.L., Slitt A., Bothun G.D. (2022). Critical new insights into the binding of poly- and perfluoroalkyl substances (PFAS) to albumin protein. Chemosphere.

[11] Kar S., Sepúlveda M.S., Roy K., Leszczynski J. (2017). Endocrine-disrupting activity of per- and polyfluoroalkyl substances: Exploring combined approaches of ligand and structure based modeling. Chemosphere.

[12] Hoover G., Kar S., Guffey S., Leszczynski J., Sepúlveda M.S. (2019). In vitro and in silico modeling of perfluoroalkyl substances mixture toxicity in an amphibian fibroblast cell line. Chemosphere.

[13] Mauri A., Bertola M. (2022). Alvascience: A new software suite for the QSAR workflow applied to the blood brain barrier permeability. Int. J. Mol. Sci..

[14] Ambure P., Roy K. DTC Lab Software Tools. http://teqip.jdvu.ac.in/QSAR_Tools/.

[15] Chicco D., Jurman G. (2023). The Matthews correlation coefficient (MCC) should replace the ROC AUC as the standard metric for assessing binary classification. BioData Min..

[16] (2023). LigPrep, Schrödinger Release 2023-2: Schrödinger, LLC, New York, NY. https://www.schrodinger.com/products/ligprep.

[17] (2023). Glide, Schrödinger Release 2023-2: Schrödinger, LLC, New York, NY. https://www.schrodinger.com/products/glide.

[18] Generalized Read-Across (GenRA). https://comptox.epa.gov/genra/.

